# The Influence of Modern Social Media on Dermatologist Selection by Patients

**DOI:** 10.7759/cureus.11822

**Published:** 2020-12-01

**Authors:** Mohammed Albeshri, Ru'aa Alharithy, Saad Altalhab, Omar B Alluhayyan, Abdulrahman M Farhat

**Affiliations:** 1 Dermatology, Unaizah College of Medicine and Medical Sciences, Qassim University, Qassim, SAU; 2 Dermatology, Security Forces Hospital, Riyadh, SAU; 3 Dermatology, Imam Mohammad Ibn Saud I University, Riyadh, SAU; 4 Medicine, Unaizah College of Medicine and Medical Sciences, Qassim University, Qassim, SAU; 5 Medicine, Sulaiman Al Rajhi University, Qassim, SAU

**Keywords:** social media, dermatologists, communication, aesthetic dermatology

## Abstract

Objective

Social media have become the platform of choice for people seeking immediate access to information. They have become so ubiquitous and pervasive that many people are using them to research health care providers and communicate with them about their issues. This study looks into this phenomenon, focusing on how it affects people’s thinking when deciding which doctor to see for skin-related concerns.

Methodology

A cross-sectional study was conducted among patients at Derma Clinic in Riyadh, Saudi Arabia. Data were collected using a validated self-administered questionnaire. A total of 365 patients were included in the analysis.

Results

Out of 365 participants, 44.9% visited the center for medical purposes, while 45.8% visited for cosmetic purposes. Sixty-six percent of the participants (n=241) went to a dermatologist they knew, and only 21% of those participants knew their dermatologist from social media (Twitter, Instagram, Snapchat, Facebook, and Telegram). About 44.54% preferred to know more about their dermatologists from Twitter, followed by Instagram 27.96%, Snapchat 24.64%, and Facebook 2.84%. A significant proportion of Instagram users came to the dermatology clinic for cosmetic purposes.

Conclusion

As per the findings of this study, we found the highest preferred way of knowing the doctor for medical or cosmetic purposes was through a friend, followed by a family member and social media. Although most of the participants still preferred to visit a dermatologist based on their friend’s recommendation, social media offer patients a platform upon which to launch their search for a dermatologist. Among social media users, patients who visit dermatology clinics for medical reasons preferred Twitter, while those who came for cosmetic reasons preferred Instagram to follow their doctors, underlining the importance of aesthetic dermatology as a visual field.

## Introduction

The first major social media network began in 1997, expanding in popularity and application from virtual non-existence to an estimated 1.43 billion users by the end of 2012 [1, 2]. An astounding 728 million persons view over 24 billion pages on Facebook daily, making it the second most popular website in the world and the United States [3]. Following closely behind, Twitter has risen to the ninth most visited website globally, with over 39 million users viewing 253 million pages daily [4]. On Instagram, approximately 75 million daily users have shared 16 billion photos to date [5]. Snapchat is a relative newcomer in the social media space, having been developed in 2011. Snapchatters watch over 10 billion videos and spend an average of 25-30 minutes per day on the application [6].

Social networking sites allow users to easily construct profiles and connect with other users with whom they share information through various integrated tools within the network, including updates, messages, pictures, and video uploads [7]. These sites also facilitate online support communities and provide engaged patients with platforms to discuss specific conditions and diseases [8]. They are emerging as a place for scientific journals to reach broader audiences and as a potential tool for education and conversation between patients and health care providers.

Apart from using social media to support patients, patient organizations are also taking advantage of these platforms as a source of patient-focused information on medical illnesses, issues, treatments, prevention, and awareness [9].

There are thousands of groups within these online communities, connecting individuals with similar interests and affiliations from all over the world [10]. Indeed, more than 40% of the health care consumers nowadays use social media for their health care information requirements [11]. Such practice is more pronounced among users between 18 and 24 years old, as compared to the 45-54 year age group. Moreover, 90% of health care consumers in the 18-24 year age group utilize and believe in medical content shared on social media [12].

Social media have offered health care professionals a unique way to overcome gaps in delivering medical services, enabling them to reach their patients more easily [13]. They have also helped improve self-care skills among patients by keeping them up to date on specific diseases, apart from providing physicians with tremendous opportunities to conduct research in their relevant fields [14]. Some physicians even say social media are among the best ways to formulate, collect, and analyze data for scientific studies meant for journal publications [15]. Our study aimed to assess social media use among patients to determine how they affect their choice of dermatologists.

## Materials and methods

Study design, setting, and participants

The research was a cross-sectional study using a self-administrated questionnaire among patients seen at the Derma Clinic in Riyadh, Saudi Arabia. The sample size was calculated using a statistical formula for a cross-sectional survey design. Following computation, we found that the minimum sample size to achieve a precision of a 5% and a 95% confidence interval was 372, of which seven had almost empty questionnaires, so the final number was 365.

Data collection methods

The participants were informed about the content and purpose of the questionnaire and were asked to fill out the questionnaire. The data collection started in September 2016 and ended in July 2017 using a self-administered interview sheet validated by three experts in the field. The questionnaire consisted of six questions. Three questions pertained to patients’ personal information (gender, the purpose of the visit, if the patients came to visit a certain doctor). Two questions were aimed at knowing more about how the patients knew of their doctors and their preferred way of gathering more information about them. Still, another question was about the social media application that the patients used to obtain more information about their dermatologist. The questionnaire was administrated in both Arabic and English languages. 

Statistical analysis

Data were first cleaned, with those missing values being removed before statistical tests were carried out. Statistical analysis was done using the SPSS 24 statistical software package (IBM Inc., Armonk, USA). Results were presented as mean frequencies and percentages for qualitative data. A chi-square test was used for comparing qualitative variables between groups. A probability value of less than or equal to 0.05 was considered statistically significant.

## Results

**Figure 1:**
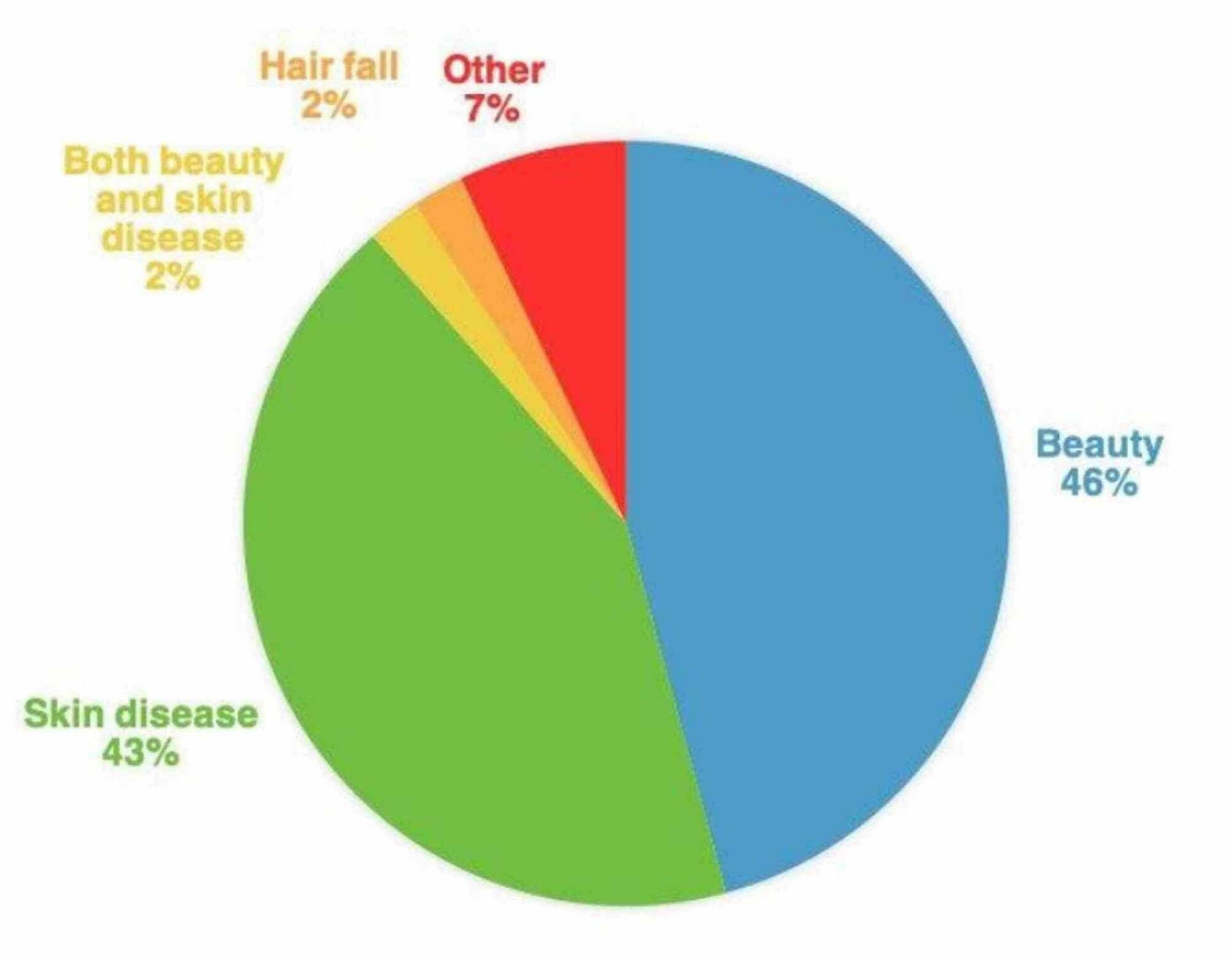
Distribution of responses about visit purpose

A total of 365 participants filled the survey, where 84.4% (n=308) were females, and 15.6% (n=57) were males. Of the participants, 44.9% (n=164) visited the dermatology clinic for medical purposes (having skin disease), 45.8% (n=167) came for cosmetic purposes, 2.2% (n=8) came for both medical and cosmetic purposes, and 7.1% (n=26) came for other reasons. Most of the patients (66%) came to a doctor they knew (Table 1 and Figure 1).

**Table 1:** The association between gender and social media knowledge and following of the dermatologist

		Gender				P-value
		Male		Female		
		N	%	N	%	
Total number of participants		57	15.6%	308	84.3%	N/A
Age mean (SD): 29.8 (10.2)	22 and younger	11	19.3%	82	26.6%	0.183
	23 to 28	11	19.3%	84	27.3%	
	29 to 35	16	28.1%	72	23.4%	
	36 and older	19	33.3%	70	22.7%	
Purpose of visit	Beauty	17	29.8%	150	48.7%	0.026
	Skin disease	35	61.4%	121	39.3%	
	Both beauty and skin disease	2	3.5%	6	1.9%	
	Hair fall	1	1.8%	7	2.3%	
	Other	2	3.5%	24	7.8%	
Particular doctor you knew	Yes	35	61.4%	206	66.9%	0.422
	No	22	38.6%	102	33.1%	
How do you know your doctor	I do not know him	3	5.3%	13	4.2%	0.549
	Previous visit	5	8.8%	31	10.1%	
	Family	22	38.6%	87	28.2%	
	Friends	17	29.8%	101	32.8%	
	Traditional media (TV – Radio – Newspaper)	0	0.0%	8	2.6%	
	New media (Twitter – Snapchat – Facebook)	10	17.5%	68	22.1%	
Preferred way to know more about your doctor is Twitter	Neutral	46	80.7%	225	73.1%	0.225
	Yes	11	19.3%	83	26.9%	
Preferred way to know more about your doctor is Instagram	Neutral	51	89.5%	255	82.8%	0.208
	Yes	6	10.5%	53	17.2%	
Preferred way to know more about your doctor is Snap	Neutral	50	87.7%	263	85.4%	0.644
	Yes	7	12.3%	45	14.6%	
Preferred way to know more about your doctor is Facebook	Neutral	52	91.2%	307	99.7%	0.000
	Yes	5	8.8%	1	0.3%	
Preferred way to know more about your doctor is Telegram	Neutral	57	100.0%	308	100.0%	N/A
	Yes	0	0.0%	0	0.0%	
Application preferred to follow my doctor on is Twitter	Neutral	25	43.9%	172	55.8%	0.095
	Yes	32	56.1%	136	44.2%	
Application preferred to follow my doctor on is Instagram	Neutral	46	80.7%	198	64.3%	0.016
	Yes	11	19.3%	110	35.7%	
Application preferred to follow my doctor on is Snap	Neutral	36	63.2%	190	61.7%	0.834
	Yes	21	36.8%	118	38.3%	
Application preferred to follow my doctor on is Facebook	Neutral	52	91.2%	301	97.7%	0.011
	Yes	5	8.8%	7	2.3%	
Application preferred to follow my doctor on is Telegram	Neutral	56	98.2%	300	97.4%	0.706
	Yes	1	1.8%	8	2.6%	

 

**Figure 2:**
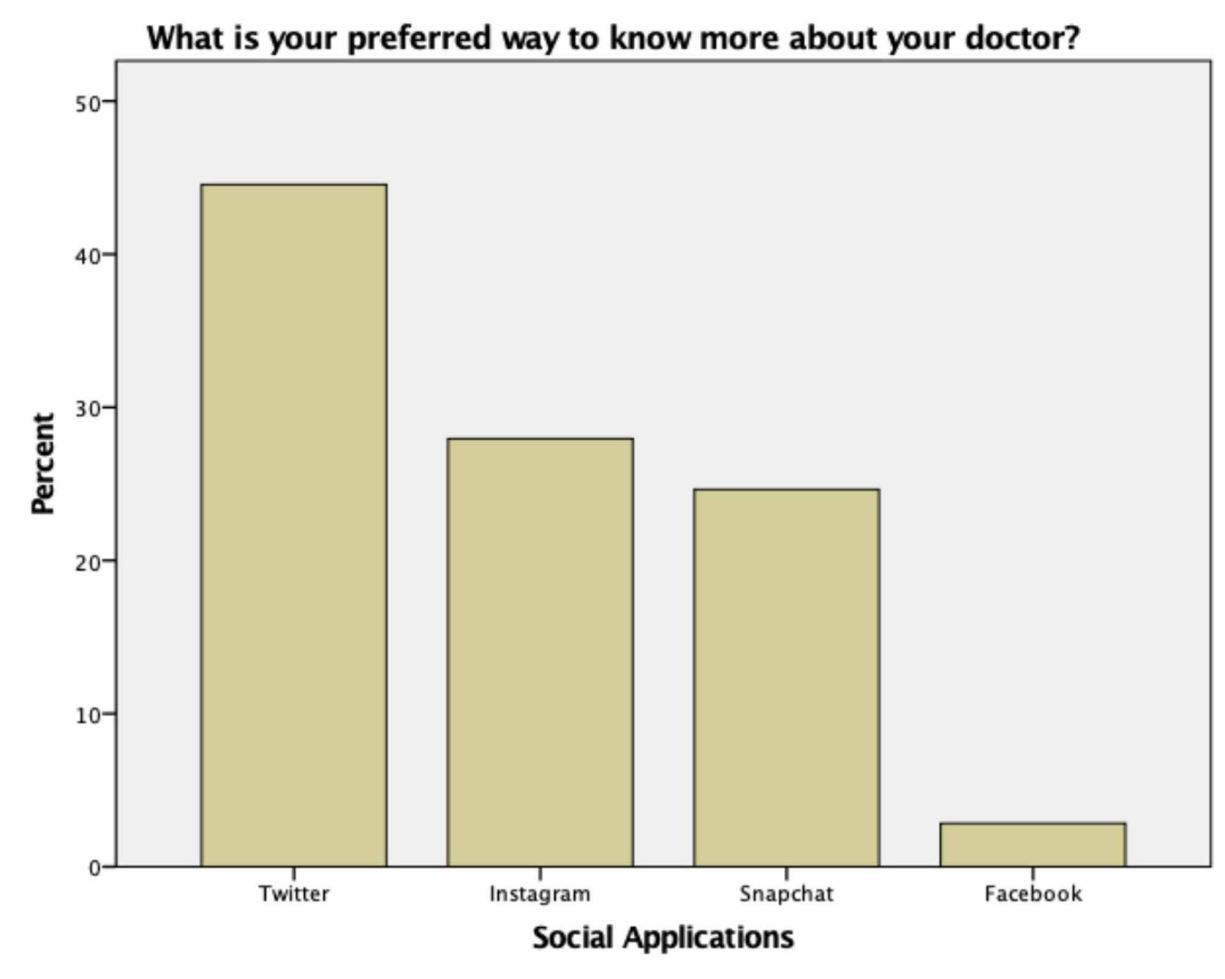
Responses on the preferred social-media applications to know more about the doctor

**Figure 3:**
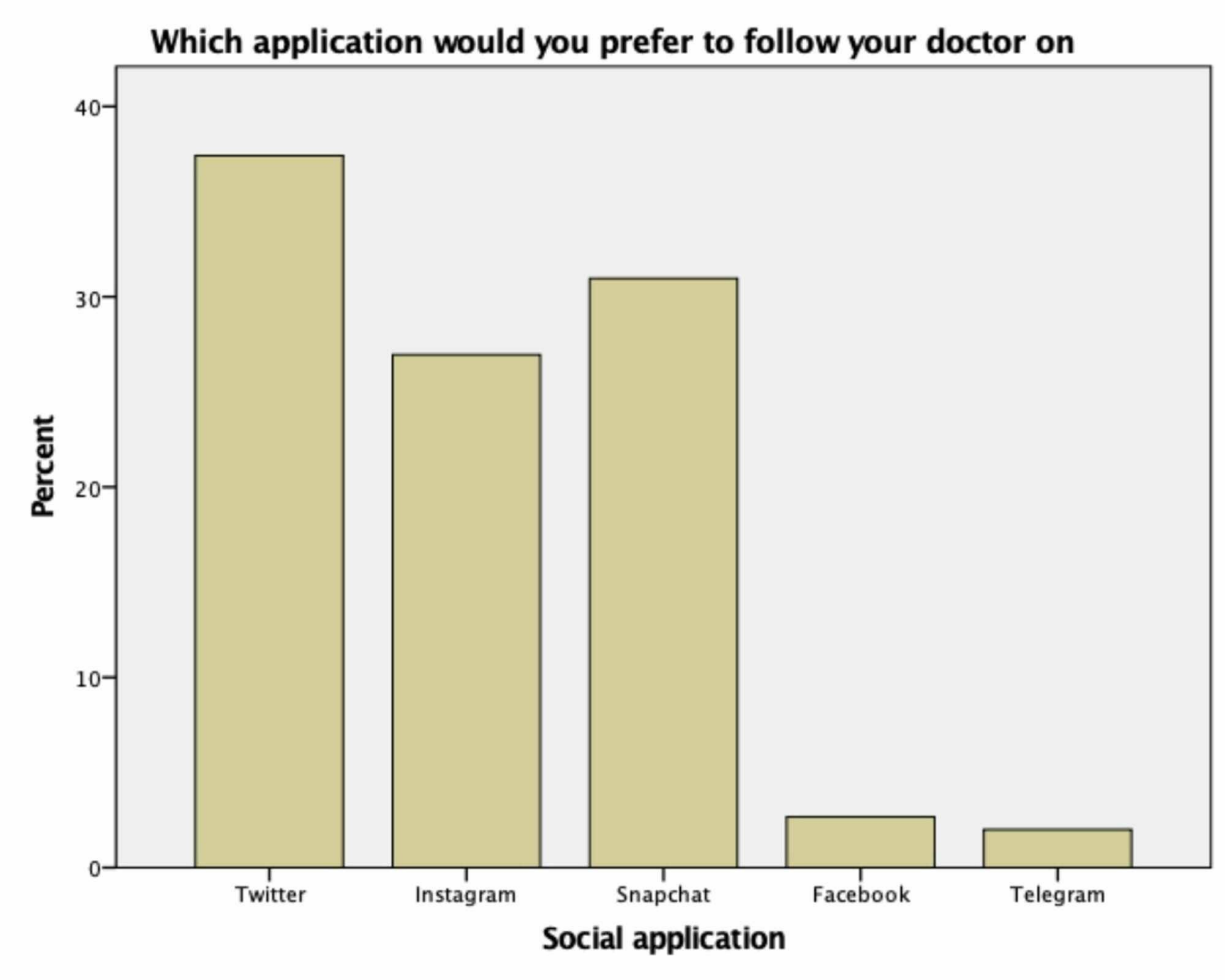
Responses on the preferred social-media applications to follow the doctor

The most common way the patients heard about their dermatologist was through friends 32.3% (n=118), followed by a family member 29.9% (n=109), then new social media (Twitter - Snapchat - Instagram - Facebook) 21.4% (n=78). Only 2.2% of participants previously knew their doctors from traditional media (newspaper - radio-TV) compared to 20.7% knew from new social media (Twitter - Snapchat - Instagram - Facebook). When participants asked about the preferred way to get more information about their doctor's reputation and performance, 94 out of the 211 (44.54%) answered Twitter, 59 (27.96%) answered Instagram, 52 (24.64%) answered Snapchat, six (2.84%) answered Facebook (Figure 2). When participants were asked about applications used to follow their doctor, 168 participants (37.4%) preferred Twitter, 121 (26.9%) preferred Instagram, 139 (31%) preferred Snapchat, 12 (2.7%) preferred Facebook, and only nine (2%) preferred Telegram. As more than one answer was allowed in these questions, we have a higher number of responses (Figure 3).

There was a statistically significant association between participants who chose Instagram as their preferred way to get more information about the doctor's reputation and performance and the purpose of visit (p=0.009), where the larger number of participants who used the application came to the dermatology clinic for a cosmetic purpose (Table 2). Moreover, there was a significant association between the purpose of the visit and participants' use of both Twitter and Instagram to follow their doctors (p=0.021 and p=0.003, respectively). Most of those who came for a medical purpose preferred to follow their dermatologist on Twitter, while those seeking cosmetic consultation or treatment preferred Instagram (Table 3).

**Table 2:** The association between the purpose of the visit and used social media for knowing and following the dermatologist

What is your preferred way to know more about your doctor?	Reason for visit	Neutral		Yes		p-value
		N	%	N	%	
Preferred way to know more about your doctor is Twitter	Beauty	125	46.1%	42	44.7%	0.071
	Skin disease	118	43.5%	38	40.4%	
	Both beauty and skin disease	8	3.0%	0	0.0%	
	Hair fall	6	2.2%	2	2.1%	
	Other	14	5.2%	12	12.8%	
Preferred way to know more about your doctor is Instagram	Beauty	129	42.2%	38	64.4%	0.009
	Skin disease	142	46.4%	14	23.7%	
	Both beauty and skin disease	8	2.6%	0	0.0%	
	Hair fall	6	2.0%	2	3.4%	
	Other	21	6.9%	5	8.5%	
Preferred way to know more about your doctor is Snap	Beauty	141	45.0%	26	50.0%	0.290
	Skin disease	136	43.5%	20	38.5%	
	Both beauty and skin disease	5	1.6%	3	5.8%	
	Hair fall	7	2.2%	1	1.9%	
	Other	24	7.7%	2	3.8%	
Preferred way to know more about your doctor is Facebook	Beauty	164	45.7%	3	50.0%	0.162
	Skin disease	154	42.9%	2	33.3%	
	Both beauty and skin disease	8	2.2%	0	0.0%	
	Hair fall	7	1.9%	1	16.7%	
	Other	26	7.2%	0	50.0%	
Preferred way to know more about your doctor is Telegram	Beauty	167	45.8%	0	0.0%	N/A
	Skin disease	156	42.7%	0	0.0%	
	Both beauty and skin disease	8	2.2%	0	0.0%	
	Hair fall	8	2.2%	0	0.0%	
	Other	26	7.1%	0	0.0%	

**Table 3:** The association between the purpose of the visit and social media preference for following the dermatologist

Which social application would you prefer to follow your doctor?	Reason for visit	Neutral		Yes		p-value
		N	%	N	%	
Application preferred to follow my doctor on is Twitter	Beauty	102	51.8%	65	38.7%	0.021
	Skin disease	75	38.1%	81	48.2%	
	Both beauty and skin disease	2	1.0%	6	3.6%	
	Hair fall	2	1.0%	6	3.6%	
	Other	16	8.1%	10	6.0%	
Application preferred to follow my doctor on is Instagram	Beauty	98	40.2%	69	57.0%	0.003
	Skin disease	122	50.0%	34	28.1%	
	Both beauty and skin disease	5	2.0%	3	2.5%	
	Hair fall	4	1.6%	4	3.3%	
	Other	15	6.1%	11	9.1%	
Application preferred to follow my doctor on is Snap	Beauty	97	42.9%	70	50.4%	0.286
	Skin disease	101	44.7%	55	39.6%	
	Both beauty and skin disease	4	1.8%	4	2.9%	
	Hair fall	4	1.8%	4	2.9%	
	Other	20	8.8%	6	4.3%	
Application preferred to follow my doctor on is Facebook	Beauty	163	46.2%	4	33.3%	0.557
	Skin disease	150	42.5%	6	50.0%	
	Both beauty and skin disease	8	2.3%	0	0.0%	
	Hair fall	7	2.0%	1	8.3%	
	Other	25	7.1%	1	8.3%	
Application preferred to follow my doctor on is Telegram	Beauty	165	46.3%	2	22.2%	0.305
	Skin disease	151	42.4%	5	55.6%	
	Both beauty and skin disease	8	2.2%	0	0.0%	
	Hair fall	8	2.2%	0	0.0%	
	Other	24	6.7%	2	22.2%	

## Discussion

This study aimed to determine how social media applications affected the clinical practice of dermatology and influenced patients’ selection of dermatologists. In recent years, social media and the internet have grown in popularity, developing into new communication tools between patients and physicians [16]. About 4% of daily searches on the internet globally are health-related [17]. Our results show that most of the participants visited the dermatology clinic for cosmetic purposes. Furthermore, a large proportion of participants chose a doctor they knew previously. The preferred way of knowing the doctor for medical or cosmetic purposes was through a friend, followed by a family member and social media.

The findings are consistent with the study by Aydin et al., which reported that the recommendation by former patients primarily dictated the choice of a physician by patients [18]. Furthermore, in a US study, a high proportion of patients preferred to seek reputable healthcare providers' services [19]​​​​​​. 

Studies on the prevalence of social media use in patient-physician communication and the effects of social media and the internet on patients’ choice of physician, hospital, or treatment options have intensified in recent research [16, 20, 21]. Our research found that the number of participants who knew about their doctors from social media (Twitter, Instagram, Snapchat, Facebook, and Telegram) was 9.7 times higher than those who knew them from traditional sources (newspaper, radio, and TV). A quantitative study conducted in Egypt by Younis et al. in 2017 reported that among 116 dermatology residents, 91.38% were into Facebook, but their most common method for interaction with patients was the telephone (61.21%) [20]**. **Our research supports this finding, as we found the use of Facebook and Telegram were less common in patient-physician communications. This can be attributed to the fact that private social networking sites are mainly based on close-friendship and friendship requests [22].

Social media offer a potentially effective and credible platform to obtain and engage new patients. Our research found a significant association regarding the use of Twitter and of Instagram applications by our participants to follow their doctors (p=0.021 and p=0.003, respectively).

The use of social media and the internet in the dermatology field is increasing. Patients can share their health-related experiences or issues via social media and in discussion forums with experienced physicians. But despite the benefits and advantages of social media for seeking medical advice or treatment, legal liability and possible risks of sharing information online should be kept in mind. There is yet much to learn about best practices for social media use by health professionals, and this is something dermatologists should look into. As in other studies, our research was limited by its cross-sectional design and small sample size.

## Conclusions

Even though social media use among dermatology patients for looking up and following their doctors was more common than the traditional media, most of the participants still preferred to visit a dermatologist based on their friend’s recommendation. Overall, those who used social media to choose and follow their doctors preferred Twitter. Among social media users, patients who visit dermatology clinics for medical reasons preferred Twitter while those who came for cosmetic reasons preferred Instagram to follow their doctors, which is understandable given that Instagram enables people to showcase their looks, influencing many to try aesthetic dermatology, a visual field. The available literature on how social media influences dermatology practice is still scarce, and further research is needed.
